# Evolutionary maintenance of genomic diversity within arbuscular mycorrhizal fungi

**DOI:** 10.1002/ece3.4834

**Published:** 2019-02-11

**Authors:** Thomas W. Scott, E. Toby Kiers, Guy A. Cooper, Miguel dos Santos, Stuart A. West

**Affiliations:** ^1^ Department of Zoology University of Oxford Oxford UK; ^2^ Institute of Ecological Sciences, Faculty of Earth and Life Sciences Vrije Universiteit Amsterdam The Netherlands; ^3^ Department of Social Psychology and Social Neuroscience, Institute of Psychology University of Bern Bern Switzerland; ^4^ Magdalen College Oxford UK

**Keywords:** arbuscular mycorrhizal fungi, chimera, genetic conflict, individuality, intraorganismal genetic heterogeneity, levels of selection, modular organisms, mosaic, mycorrhizal networks, organismality

## Abstract

Most organisms are built from a single genome. In striking contrast, arbuscular mycorrhizal fungi appear to maintain genomic variation within an individual fungal network. Arbuscular mycorrhizal fungi dwell in the soil, form mutualistic networks with plants, and bear multiple, potentially genetically diverse nuclei within a network. We explore, from a theoretical perspective, why such genetic diversity might be maintained within individuals. We consider selection acting within and between individual fungal networks. We show that genetic diversity could provide a benefit at the level of the individual, by improving growth in variable environments, and that this can stabilize genetic diversity even in the presence of nuclear conflict. Arbuscular mycorrhizal fungi complicate our understanding of organismality, but our findings offer a way of understanding such biological anomalies.

## 1 INTRODUCTION

Most organisms are built from a single collection of genes (*genome*), copied into all nuclei, across all cells. Genomic homogeneity means that the cells and nuclei within organisms have the same evolutionary interest, to transmit that genome to the next generation (Buss, 1988; Maynard Smith & Szathmáry, 1997; Strassmann & Queller, 2004). The components of organisms therefore work together, cooperatively, to increase reproductive success. From an evolutionary perspective, this cooperation and lack of conflict define organisms (Maynard Smith & Szathmáry, 1997; Queller & Strassmann, 2009, 2016; West, Fisher, Gardner, & Kiers, 2015).

Arbuscular mycorrhizal (AM) fungi appear to be a striking exception to this rule of genomic homogeneity within organisms (Angelard, Colard, Niculita‐Hirzel, Croll, & Sanders, 2010; Angelard et al., 2013; Ehinger, Croll, Koch, & Sanders, 2012; Wyss, Masclaux, Rosikiewicz, Pagni, & Sanders, 2016). AM fungi form large branching networks composed of filaments called hyphae. These hyphal networks (*individuals*), which germinate from spores, live in soil and colonize plant roots, exchanging mineral resources for host carbon (Bonfante & Genre, 2010). A hyphal network can potentially bear thousands of coexisting nuclei at once (*heterokaryotic*) (Sanders & Croll, 2010), and connect multiple plants simultaneously (Rosendahl & Stukenbrock, 2004). There are no internal septal walls within the hyphal networks (*coenocytic*), and so nuclei can potentially move across entire networks. Individual networks of closely related fungal strains can fuse (*anastomose*) and share nuclei (Giovannetti, Avio, & Sbrana, 2015), potentially generating individuals bearing two genomes (Corradi & Brachmann, 2017; Ropars et al., 2016) or possibly many more (Croll et al., 2008; de Novais, Sbrana, Júnior, Siqueira, & Giovannetti, 2013; Hijri & Sanders, 2005; Kuhn, Hijri, & Sanders, 2001; Sanders & Croll, 2010; Wyss et al., 2016). Small levels of genomic variation might also arise through different de novo mutations occurring in different nuclei within an individual (Tisserant et al., 2013). When individuals sporulate, hundreds of nuclei flow into the emerging spore, allowing a large portion of the genomic variation to be maintained (Jany & Pawlowska, 2010).

From an evolutionary perspective, the potential for genomic variation within individuals, and the apparent absence of any mechanism to regulate it, poses problems (Frank, 1995, 2003; Strassmann & Queller, 2007). First, it is likely that nuclei replicate at different rates within hyphal networks (Jany & Pawlowska, 2010; Roberts & Gladfelter, 2015), so we would expect the most competitive and fast‐growing nucleus lineage to outcompete the rest. In other words, we would expect *within‐individual selection* to lead to genomic purity (Gilbert, Foster, Mehdiabadi, Strassmann, & Queller, 2007; Inglis, Ryu, Asikhia, Strassmann, & Queller, 2017; Kooij, Aanen, Schiøtt, & Boomsma, 2015; Meunier, Hosseini, Heidari, Maryush, & Johannesson, 2018; Vreeburg, Nygren, & Aanen, 2016). Within‐individual evolution would eventually lead to genomic purity even if nuclei are equally competitive, through drift, because not all nuclei migrate from parent hyphal networks into daughter cells (Angelard et al., 2010; Boon, Zimmerman, St‐Arnaud, & Hijri, 2013; Marleau, Dalpé, St‐Arnaud, & Hijri, 2011; Masclaux, Wyss, Mateus‐Gonzalez, Aletti, & Sanders, 2018). Secondly, we would expect genomic variation within individuals to lead to conflict among different genomic (nuclear) lineages and hence reduce the fitness of that individual. Consequently, individuals with high genomic variation could be outcompeted by individuals with genomic homogeneity. In other words, we would expect *between‐individual selection* to also lead to genomic purity (Bastiaans, Debets, & Aanen, 2016; Meunier et al., 2018).

We address the theoretical problem of why genomic diversity would be maintained in AM fungi. We develop theoretical models to address two questions. First, can genomic diversity provide a benefit at the individual level that gives individuals with genomic diversity a competitive advantage over those with genomic homogeneity, despite potential conflict between genomes? Second, how can genomic diversity be maintained within individuals, if one nucleus lineage is more competitive and able to reproduce faster? Our hypothesis is that different fungal genotypes are better at colonizing different plant species, and so fungal individuals with genomic diversity are better able to better colonize multiple plants. If fungal individuals encounter sufficiently different plant species, then this could maintain genomic diversity.

We develop simple analytical models, building upon previous theory, to illustrate the general points. We then develop a more detailed individual‐based simulation, to better match the biology of AM fungi. To emphasize applicability to other organisms, we use the general terms “individual” and “genomic diversity,” rather than the AM‐specific terms “hyphal network” and “nuclear diversity.” Conversely, although we often talk specifically about competing nucleus lineages, our theory applies more generally to *genomic lineages* of a modular organism that may in fact be cell lines as opposed to nucleus lines (Pineda‐Krch & Lehtila, 2004; Strassmann & Queller, 2004). The extent of genomic diversity in AM fungi is a matter of considerable debate, which is beyond the scope of our paper (Lin et al., 2014; Maeda et al., 2018; Ropars & Corradi, 2015; Tisserant et al., 2013; Wyss et al., 2016). Our aim is to examine how, if diversity exists, it could plausibly be maintained (Bruns, Corradi, Redecker, Taylor, & Öpik, 2017; Sanders, 2018).

## 2 MODELS

### 2.1 Competing individuals

Our first question is whether genomic diversity can provide a benefit at the level of the individual, allowing individuals with genomic diversity to outcompete those without. Our hypothesis is that genomic diversity provides a way of acquiring a generalist phenotype, which is better able to cope with an unpredictable environment. We take an ESS approach, based on previous theory (Levins, 1962), to find the level of genomic diversity that maximizes individual fitness.

We assume that there are two different plant species, which we term plant 1 (*P_1_*) and plant 2 (*P_2_*). Individual hyphal networks associate with and grow on multiple plants simultaneously. We assume that all individuals are in the same environment, with a proportion *p* of their interactions being with plant 1 (*P_1_*), and the remaining proportion (1–*p*) with plant 2 (*P_2_*). The overall fitness of an individual (*W*) depends on its fitness (how well it grows) on type 1 plants (*w_1_*), weighted by the extent to which it is growing on type 1 plants (*p*), and its fitness on type 2 plants (*w_2_*), weighted by the extent to which it is growing on type two plants (1–*p*), with *W* = *pw_1_* + (1–*p*)*w_2_*. This equation was originally formulated as a general way to represent fitness under simultaneous exposure to two different environments (Levins, 1962). For our purposes, the two plant hosts provide the two environments.

We make the fitness terms in Levins’ equation (*w_1_* and *w_2_*) explicit, so that the fitness of an individual can be written:

(1) $$W\left(x\right)=p\left(\kappa +\left(1-\kappa \right)x^{\alpha }\right)+\left(1-p\right)\left(\kappa +\left(1-\kappa \right){\left(1-x\right)}^{\alpha }\right).$$

Individuals contain two types of nuclei (*N_1_* and *N_2_*), which are genetically distinct, nonrecombining, and each specialized on one plant type, *N_1_* on *P_1_*, and *N_2_* on *P_2_* (Chen et al., 2018b). Fitness on each plant depends on the parameter *x*, which is the individual's proportion of type 1 nuclei (*N_1_*) relative to type 2 nuclei (*N_2_*). There is a trade‐off, meaning as the type 1 nuclear proportion *x* is increased, fitness on *P_1_* (*w_1_*) increases from κ to 1, but fitness on *P_2_* (*w_2_*) decreases, symmetrically, from 1 to κ. The slope of fitness (*w_1_*, *w_2_*) against nucleus proportion (*x*) may be concave (0 < *α* <  1), corresponding to diminishing fitness returns to plant specialization, or convex (*α* > 1), corresponding to accelerating returns.

The curvature parameter α encapsulates multiple biological phenomena. If the size of the hyphal network (individual) is large relative to the number of plant associations it has, there may be an overabundance of nuclei in the network (Shoji, Kikuma, Arioka, & Kitamoto, 2010). This would make specialized nuclei less effective at high proportions, where they are not being fully utilized, causing diminishing returns to specialization (0 < *α* < 1). Conversely, small networks with relatively many plant associations may be insufficiently productive to engage each of their host plants in a mutually beneficial relationship, given that host plants divert their resources away from poorly cooperating AM fungi (Kiers et al., 2011). This would render specialized nuclei ineffective at low proportions, causing increasing returns to specialization (*α* > 1). Conflict and interference between nuclei would also lead to increasing returns from specialization. Nuclear conflict could render specialized nuclei ineffective at low proportions where their relatedness to other nuclei is low. Interference among nuclei may mean low proportions of specialized nuclei are swamped and unable to contribute to network‐level functionality.

We now ask when genomic diversity (0 < *x* < 1), as opposed to purity (*x* = 0 or *x* = 1), is favored at the individual level. This will be the case when the fitness of an individual (*W*; Equation 1) is maximized at some intermediate nuclear proportion, which requires the mathematical conditions: $\frac{\mathrm{dW}}{\mathrm{dx}}=0,\;\frac{d^{2}W}{dx^{2}}<0,0<x^{*}<1$ (Maynard Smith & Price, 1973; Taylor, 1996). These conditions are satisfied when there is a mixture of the two plant species in the environment (0 < *p* < 1), and the returns to specialization are diminishing (0 < α < 1) (Appendix 1). Given this, genomic diversity is favored, and the specific nuclear proportion (*x*) that is favored is as follows:

(2) $$x^{*}=\frac{1}{1+{\left(\frac{1-p}{p}\right)}^{\frac{1}{1-\alpha }}}.$$

**Figure 1:**
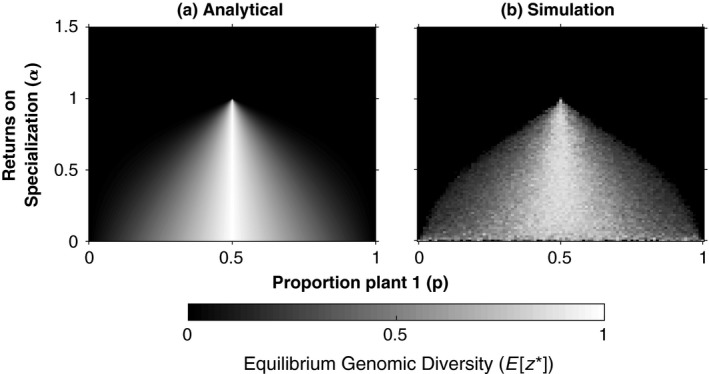
Effect of environmental variability (*p*) and the curvature of specialization returns (*α*) on genomic diversity. Both parts show the level of genomic diversity at evolutionary equilibrium (*E*[z*]) in the absence of nuclear replicative differences. The y‐axis is the shape of the relationship between fitness and nucleus proportion (*α*), where *α* > 1 reflects accelerating returns to specialization and *α* < 1 reflects diminishing returns. The *x*‐axis is the proportion of plant species one (*p*), relative to plant species two (*1‐p*). Part (a) shows the analytically derived ESS of the Competing Individuals model, and part (b) shows the results of our individual‐based simulation (*n* = 2000, *f* = 0.005, *d* = 0.5, *m* = 0). The results of our ESS model and our simulation are quantitatively equivalent, showing that genomic diversity is stabilized, for diminishing returns to specialization (*α* → 0) and mixed environments (*p* → 0.5), in the absence of replicative differences between nuclei

We can convert the equilibrium nuclear proportion (x*) to a measure of genomic diversity (z*), which ranges from zero to one, and is maximal when there is an equal proportion of type 1 and type 2 nuclei (z* = 1–2|x*‐0.5|). More extreme genomic diversity is favored by between‐individual selection (z*→1) as returns become more diminishing (α → 0) and the environment becomes more mixed (*p* → 0.5) (Figure 1a). As returns become more diminishing, the relative benefit of having a small fraction of each nucleus is increased, favoring diversity. Our result illustrates, for the specific case of genomic diversity in an individual, how life history and ecology can select for “generalist” phenotypes (Hedrick, Ginevan, & Ewing, 1976; Levins, 1962, 1966; Levins & MacArthur, 1966). Furthermore, our model implies that genomic diversity might be favored in some, but not all environments (Sanders, 2018). Discrepancies between different empirical estimates of genomic diversity in natural AM fungi populations might reflect environmental differences in either: (a) the density of plants (which may affect the returns on nucleus specialization); or (b) the mixture of different plant types.

### 2.2 Competing nuclei

Our above model examined why individuals with genomic diversity might outcompete individuals with genomic homogeneity. A potential problem here is that nucleus (genome) lineages might be more competitive or selfish, replicating faster within individuals and eliminating genomic diversity as they come to dominance (Frank, 1998). Consequently, we now examine whether such within‐individual competition could be balanced by the benefits of being in an individual with genomic diversity (between‐individual selection). We are therefore taking the result from the Competing Individuals (Levins, 1962) model that individuals with genomic diversity have a higher fitness, and examining the consequences for the maintenance of within‐individual genomic diversity. Our aim here is to analyze an abstract, heuristic case—in the following section, we use a simulation approach to analyze a more biologically realistic scenario.

We model a population of individuals assuming different proportions of type 1 (*N_1_*) relative to type 2 (*N_2_*) nuclei, *x*. We model the population as a distribution with a mean nuclear proportion *E*[*X*]. Every generation, individuals undergo nucleus replication, where *within‐individual* selection can occur, then asexual reproduction (sporulation), where *between‐individual* selection can occur (Supporting information Figure S3). There is no sharing of nuclei between individuals; individuals die at an arbitrary rate independent of nuclear proportion (*x*); offspring have the same nuclear proportion (*x*) as their asexual parent (perfect inheritance).

In the nucleus replication phase, type 1 and type 2 nuclei replicate and compete within individuals, with type 1 nuclei gaining a propagative advantage. We assume a competitive regime within individuals in which the population average nuclear proportion increases by some constant value (*θ*, where *θ* > 0). Individuals then reproduce (sporulate) asexually in proportion to their (individual) fitness. The fitness of an individual increases as its genomic diversity approaches some environmentally determined optimal value (*μ*, where 0 < *μ* < 1). We assume an abstract competitive regime, contingent on the exact form of the distribution of individuals, and of fitness, across different nuclear proportions, in which the response of the population to between‐individual selection is constant and given by *s* (0 < *s* < 1). This will be higher if nuclei strongly affect fitness, and if there is high variation between individuals. Combining our assumptions, the generational change in mean nuclear proportion is as follows:

(3) $$E{\left[X\right]}_{t+1}=s\mu +\left(1-s\right)\left(E{\left[X\right]}_{t}+\theta \right).$$

We set *E*[*X*]*_t_* = *E*[*X*]*_t_*_ + 1_ = *E*[X*], and find that the equilibrium (*absorption*) state of the distribution occurs at a mean genomic diversity of $E\left[X^{*}\right]=\mu +\frac{1-s}{s}\theta .$ We show in Appendix 2 that this state corresponds to genomic diversity (0<*E*[X*]<1) when:

(4) s(1-μ)>(1-s)θ.

The left‐hand side *s*(1–*μ*) represents the stabilizing force of between‐individual selection, effective when between‐individual selection strongly disfavors fast‐replicating nuclei (high *s*; low *μ*). The right‐hand side (1–*s*)*θ* represents the destabilizing, directional force of within‐individual selection, effective when competitive differences between nuclei within individuals are large relative to the competitive differences between individuals (high *θ*; low *s*). Genomic diversity is evolutionarily stabilized if between‐individual selection for genomic diversity exceeds within‐individual selection for competitive genomes (nuclei).

This condition is analogous to mutation‐selection balance in population genetics (Haldane, 1927; Lande, 1975), and group versus individual selection in social evolution theory (Hamilton, 1975; Price, 1972). In these cases, a given evolutionary outcome is dependent on how two opposing evolutionary forces are resolved (Frank, 2011). This perspective provides a framework for understanding why genomic diversity is common in organisms that enforce synchronous nuclear replication (*θ* = 0), and why nonfunctional “cheating” nuclei are sometimes evolutionarily stable (Appendix 3). Our qualitative conclusions hold when the order of within‐ and between‐individual selection is reversed (Supporting information Data S1), when within‐individual selection and between‐individual selection are modeled in a more general, less abstracted, framework (Supporting information Data S2), and when an explicit form of the distribution of individuals is assumed (*unpublished*).

### 2.3 AM fungi simulation

In the Competing Individuals model, we showed that between‐individual selection can favor within‐individual genomic diversity. In the Competing Nuclei model, we took this result and showed that diversity can be stably maintained even if genomes compete within individuals. However, to make our analysis general and analytically tractable, we made several simplifying assumptions with regard to: within‐individual selection (nuclear replication was not explicitly modeled); between‐individual selection (distribution of individuals, and of fitness, across different nuclear proportions, was not explicitly modeled); unstructured populations (no dispersal); no fusion of individuals (*anastomosis*); no stochasticity regarding which nuclei enter asexual spores (perfect inheritance of the nuclear proportion, *x*).

**Figure 2:**
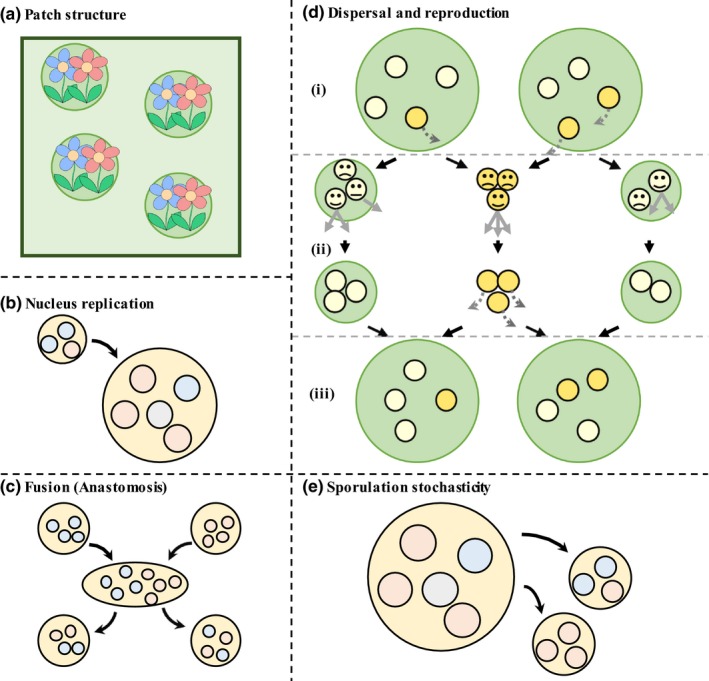
Simulation lifecycle. (a) The population of individuals (green box) is patch structured (circles containing plants). (b) Type 1 nuclei (red) replicate faster than type 2 nuclei (blue). (c) Fusion (*anastomosis*) is pairwise, with nuclei shared evenly between individuals via the formation then lesion of a large fused individual. (d) Individuals with dispersing offspring are orange, and compete with each other globally. Individuals with non‐dispersing offspring are beige, and compete with each other locally on their native patch (green circles). (dii) Individuals with higher fitness (smile) are more likely to reproduce (gray solid lines) into free spots. (diii) Offspring that have dispersed (orange) are sorted at random back into patches (green circles). (e) An offspring's genotype deviates stochastically from its asexual parent's genotype

We built a simulation model that allowed us to relax these simplifying assumptions, resulting in a closer representation of the biology of AM fungi and many other modular organisms (Figure 2). We have two broad aims with our simulation. First, we examine whether the predictions of our simple analytical models hold when more biological realism is incorporated, in a fully dynamical model. Second, we examine the influence of a number of additional factors, including differential rates of replication between strains, the fusion of individuals (*anastomosis*), dispersal, and spore size.

#### 2.3.1 Simulation details

We implement a population of *n* individuals in an individual‐based computer simulation model. The population is split into *j* patches with *n/j* individuals per patch. Individuals bear some proportion of type 1 (*N_1_*) relative to type 2 (*N_2_*) nuclei (*x*, as in previous models). An individual's initial nuclear proportion is drawn at random from a uniform distribution bound between zero and one. We assume the following lifecycle. First, individuals grow from a single spore and their nuclei grow exponentially, with type 1 nuclei replicating faster than type 2 nuclei (*r_1_* > *r_2_*). Next, individuals temporarily fuse with a random patch‐mate with some probability (*m*), share nuclei, and acquire new nuclear proportions (*x*) that are a mean of their nuclear proportions prior to fusion. The actual probability of nonself fusion between AM fungi networks in nature is unclear, with experimental estimates ranging from 6% to 90% (Giovannetti et al., 2015).

Next, individuals reproduce with a probability proportional to their fitness, which is given by Equation (1). As shown in the Competing Individuals model, this fitness equation favors genomic diversity if there is a mixture of host plants (0 < *p* < 1) and functional synergy between type 1 and type 2 nuclei (0 < *α* < 1); it favors purity of one nucleus strain otherwise. Fitness is judged relative to patch‐mates if an individual's offspring are not dispersed; fitness is judged relative to global dispersers if an individual's offspring are dispersed. Offspring dispersal occurs with some probability (d), and in AM fungi, it is likely to occur via soil‐disrupting vertebrates that transfer spores between otherwise‐isolated clusters of plants (Savary, Masclaux, et al., 2018; Vályi, Mardhiah, Rillig, & Hempel, 2016).

Offspring inherit a random sample of nuclei from their asexual parent. Offspring nuclear proportion deviates from their asexual parent by some number drawn randomly from a truncated normal distribution with a standard deviation (*f*) reflecting the level of sporulation stochasticity. The parameter *f* captures spore size—spores that inherit a small proportion of parental nuclei will be subject to higher stochasticity in nuclear inheritance (*f*). Parents die after reproducing. Though generational death (nonoverlapping generations) does not strictly apply, this is a standard modeling assumption to simplify analysis. More precise simulation details are given in Appendix 4.

We track nuclear proportion in each individual (*x*), over many generations, until the system equilibrates, to see if genomic diversity is stable. An intermediate mean nuclear proportion (0 < E[x*] < 1) is not sufficient to show that diversity is present within individuals, because this condition is also satisfied by populations comprising genomically pure individuals, some bearing type 1 nuclei and others type 2. Therefore, for each individual, we convert the nuclear proportion (*x*) to a genomic diversity score (*z*), which ranges from zero to one (*z* = 1–2|*x*–0.5|). Genomic diversity is stable if the population average level of diversity is greater than zero at equilibrium (*E*[z*]>0).

#### 2.3.2 Simulation results

**Figure 3:**
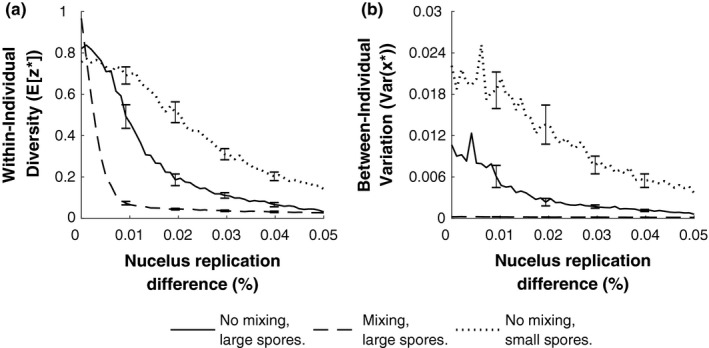
Nuclear diversity within and between individuals. The within‐individual genomic diversity (a), and between‐individual variation in nuclear proportion (b), is plotted against the nuclear replicative advantage of type 1 nuclei (*r_1_*–*r_2_*/*r_2_*) (*α* = 0.8, *p* = 0.5, *d* = 0.5, *r_2_* = 0.3, *r_1_* is varied). The different lines represent different degrees of fusion (no fusion *m* = 0; fusion: *m* = 0.05) and different spore sizes (large: *f* = 0.005; small: *f* = 0.01). Fusion between lines (higher *m*) leads to an effectively complete loss of variation between individuals, which reduces the strength of between‐individual selection, and hence leads to a faster rate of loss of within‐individual genomic diversity. Smaller spores (higher *f* = 0.01) lead to an increased sporulation stochasticity, which increases between‐individual variation, resulting in a slower rate of loss of within‐individual genomic diversity. The plots represent the average results taken across 10 trials. Error bars, where plotted, show one standard deviation above and below the mean across these 10 trials

**Figure 4:**
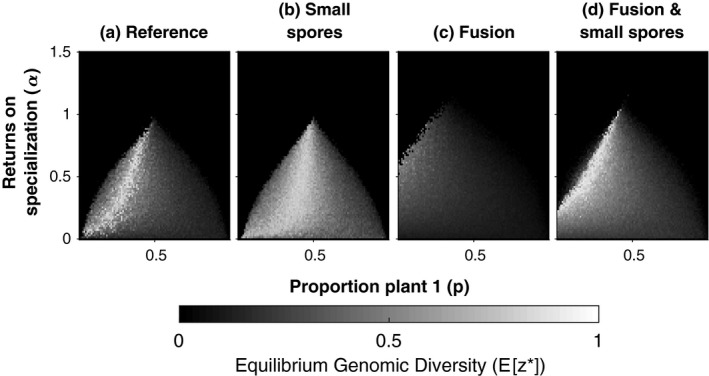
Maintenance of genomic diversity for different between‐individual selection pressures. The results of the AM Fungi Simulation model are plotted, showing the level of genomic diversity maintained within individuals at equilibrium (*E*[z*]). The heat maps plot the full range of between‐individual selection, from decelerating to accelerating returns on plant specialization (*α*, *y*‐axis), and from a plant 2 to a plant 1 dominated environment (*p*, *x*‐axis). Nucleus 1 has a replicative advantage (*r*_1_ = 0.305, *r*_2_ = 0.3), meaning (a) genomic diversity is favored in environments that are slightly dominated by plant 2, which the slower replicating nucleus is specialized on (*m* = 0, *f* = 0.005). (b) As sporulation stochasticity is increased (small spores), more genomic diversity is stable across the between‐individual selection parameter space (*f* = 0.01). (c) Fusion of individuals destabilizes genomic diversity over most of the parameter space at equilibrium (*m* = 0.05). (d) The counteracting effects of fusion and sporulation stochasticity can cancel each other out (*f* = 0.01, *m* = 0.05). These results assumed *n* = 2,000 (population size), *d* = 0.5 (dispersal)

We found broad support between our analytical models and our simulation—when there is replicative synchrony between nuclei (*r_1_* = *r_2_*), genomic diversity can be favored (Figure 1). As the replicative advantage of type 1 nuclei ((*r*_1_–*r*_2_)/*r*_2_) is increased, the diversity at equilibrium (*E*[z*]) is reduced and tends toward zero (Figure 3a; solid line). This result holds regardless of the nature of between‐individual selection (α > 0, 0 ≤ *p* ≤ 1) (Figure 4a).

Examining the extra factors in our simulation, we found that, as the replicative advantage of type 1 nuclei is increased ((*r*_1_–*r*_2_)/*r*_2_), the corresponding reduction in equilibrium genomic diversity (*E*[z*]) is exaggerated by fusion between individuals (*anastomosis*) (Figure 3a; dashed line), and attenuated by sporulation stochasticity (*f*) (Figure 3a; dotted line). The exaggerating force of fusion and the attenuating force of sporulation stochasticity are observable across the full range of between‐individual selection (α > 0, 0 ≤ *p *≤ 1) (Figure 4b and c). These effects arise because fusion reduces (Figure 3b; dashed line) between‐individual variation (Var(*x*)) and sporulation stochasticity increases it (Figure 3b; dotted line), correspondingly decreasing, and respectively, increasing, the efficacy of (stabilizing) between‐individual selection relative to (destabilizing) within‐individual selection.

We find that if genomic diversity is neutral at the within‐individual (*r*_1_ = *r*_2_) and not favored at the individual level (*α* ≥ 1), fusion (*anastomosis*) can prolong the maintenance of genomic diversity in a nonequilibrium state, by attenuating the loss of genomic diversity through individual‐level drift (Supporting information Figure S4; Bever & Wang, 2005; Pawlowska & Taylor, 2004). We find that dispersal does not significantly increase between‐individual variation (Supporting information Figure S5b), but increases the effective population size by connecting patches, in turn increasing the efficacy of between‐individual selection, slightly stabilizing genomic diversity (Supporting information Figure S5).

## 3 DISCUSSION

We provide an evolutionary explanation for the maintenance of genomic diversity in AM hyphal networks that may apply more broadly to other modular organisms. If nuclei, or specifically, *particular genes on nuclei*, are functionally specialized on different plant hosts, the cost of genome conflict borne by individuals with genomic diversity may be outweighed by the benefit of being a good generalist in a variable environment. If this between‐individual selection for genomic diversity exceeds within‐individual selection for the single fastest replicating nucleus genome, genomic diversity can be evolutionarily stable.

A key assumption in our models is that genomes (nuclei) are functionally specialized on aspects of their environment (host plants) (Strassmann & Queller, 2004). Consistent with this, the fitness of AM fungal individuals (hyphal networks) has been empirically shown to depend on an interaction between the strain of the hyphal network (genotype) and its host plant species (environment), implying nucleus specialization (Angelard et al., 2010, 2013; Ehinger, Koch, & Sanders, 2009; Savary, Masclaux, et al., 2018; Savary, Villard, & Sanders, 2018). Our model could be extended in numerous ways, to explore other factors, potentially important to AM fungi, or other organisms. For example, more nucleus types could be considered, or replication rates could be allowed to evolve (Czárán, Hoekstra, & Aanen, 2014; Frank, 1994; Wyss et al., 2016).

There are organisms other than AM fungi capable of genomic diversity, mostly restricted to those that grow through iterations of modules, like hyphae or stems, that each retains reproductive capability. These *modular* organisms include many filamentous fungi, colonial invertebrates like sponges, and plants that grow from underground connected stems called rhizomes (Herron, Rashidi, Shelton, & Driscoll, 2013; Pineda‐Krch & Lehtila, 2004). Our theory is that genomic diversity allows modular organisms to adapt to heterogeneous environments. Although a benefit to genomic diversity has been demonstrated in some other organisms, including ascidians, red algae, and other fungi, it is unclear whether environmental specialization of genomes contributes to these benefits (Rinkevich & Shapira, 1999; Santelices et al.., 1999). Other hypotheses for the benefit of genomic diversity include the following: the simultaneous generation of multiple predator‐defense phenotypes (genetic mosaic hypothesis), a mechanism for screening and selecting the best mutations, and the increased size that can result from fusing individuals (Aanen, Debets, Visser, & Hoekstra, 2008; Bastiaans, Debets, & Aanen, 2015; Castillo, Switz, Foster, Queller, & Strassmann, 2005; Foster, Fortunato, Strassmann, & Queller, 2002; Gill, Chao, Perkins, & Wolf, 1995; Jany & Pawlowska, 2010; Otto & Hastings, 1998; Otto & Orive, 1995; Whitham & Slobodchikoff, 1981).

We have focused on long‐term evolutionary (*ultimate*) causes of genomic diversity, which complement previous studies of immediate (*proximate*) generators of genomic diversity. Fusion (*anastomosis*) promotes the (proximate) spread of new genomes through populations of individuals (Croll et al., 2009; de Novais et al., 2013), but destabilizes genomic diversity in evolutionary time by reducing variation between individuals for selection to act on. Stochasticity in sporulation can result in the (proximate) loss of genomic diversity over a generation (Angelard et al., 2010; Boon et al., 2013; Marleau et al., 2011; Masclaux et al., 2018), though it stabilizes genomic diversity in evolutionary time by increasing variation between individuals. Other possible proximate influencers of genomic diversity include the following: de novo mutations; the restriction of fusion to close kin (*allorecognition*) (Czárán et al., 2014); and genetic exchange between nuclei (Chen et al., 2018a; Croll & Sanders, 2009).

To conclude, throughout this paper, we have referred to AM fungi and other modular organisms exhibiting genomic diversity as “individuals.” However, from an evolutionary perspective, “individuality” or “organismality” requires cooperation and lack of conflict between component parts (Buss, 1988; Gardner & Grafen, 2009; Maynard Smith & Szathmáry, 1997; Queller & Strassmann, 2009, 2016; West et al., 2015). Genomic conflict pulls entities away from optimal trait values (Competing Nuclei model), limiting adaptation (Strassmann & Queller, 2007). Despite this, we have shown that entities with genomic diversity can be selected and come to dominate populations. For this reason, although we may not wish to call them “organisms” (Folse & Roughgarden, 2010; Queller & Strassmann, 2009), such entities are capable of lasting evolutionary stability—hundreds of millions of years in the case of AM fungi (Heckman, 2001).

## CONFLICT OF INTEREST

None declared.

## AUTHOR CONTRIBUTION

T.W.S., E.T.K., and S.A.W. designed the study and wrote the article; T.W.S., M.d.S., and G.A.C. contributed to mathematical modeling.

## Data Availability

We agree to deposit our data to a public repository.

## APPENDIX 1

### APPENDIX 1: BETWEEN‐INDIVIDUAL SELECTION

Equation 2 gives the nuclear proportion corresponding to a stationary point (*x**). It can be seen by inspection of Equation 2 that there is always, and only, one sensical *x** value (one that lies in the range 0 ≤ *x *≤* *1) for any combination of parameter values. The stationary point (*x**) could represent a maximum (if $\frac{d^{2}W}{dx^{2}}<0$), minimum ($\frac{d^{2}W}{dx^{2}}>0$) or inflection point (if $\frac{d^{2}W}{dx^{2}}=0$). If it is a maximum, then *x** represents the individual‐favoured nuclear proportion (the ESS). If it is a minimum or inflection point, then the individual‐favoured nuclear proportion (the ESS) will be found at a boundary of *x *=* *0 or *x *=* *1.

We examine the form of the stationary point for the different ranges of the shape parameter α and baseline fitness κ. When there are increasing returns to specialisation (α > 1) and nuclear proportions affect fitness (0 ≤ κ < 1), substituting α>1 into $\frac{d^{2}W}{dx^{2}}>0$ (the condition for *x** to be a minimum) gives *px* ^α−2^ + (1 − *p*)(1 − *x*)^α−2^ > 0, which, given that 0 ≤ *p *≤* *1, is always true. *x** therefore always represents a minimum when returns are increasing. When returns to specialisation are linear (α = 1, 0≤κ<1) we find that $\frac{d^{2}W}{dx^{2}}=0$, and so *x** always represents an inflection point.

We ask what ESS will arise when *x** represents a minimum or inflection point. Given that there is only one equilibrium solution *x** for each set of parameter values, it must be the case that *W*(*x*) is maximal at either *x *=* *0 or *x *=* *1. It is maximal at *x *=* *1 if *W*(*x *=* *0)<*W*(*x *=* *1) is satisfied. Evaluating this shows that this is true for *p *>* *0.5. Conversely, *W*(*x*) is maximised at *x *=* *0 when *p *<* *0.5. When *W*(*x *=* *0)=*W*(*x *=* *1), which is the case when *p *=* *0.5, individuals can maximize fitness with either of two strategies, and individuals may assume either *x *=* *0 or *x *=* *1 at equilibrium. So, when returns to specialisation are increasing or linear (α ≥ 1), the ESS is positioned at nuclear purity, and the nucleus type that is chosen is the one that grows better with the most common plant host. In the special case where nuclear proportions have no effect on fitness (κ = 1), substitution of κ = 1 gives $\frac{d^{2}W}{dx^{2}}=0$ and *W*(*x *=* *0)=*W*(*x *=* *1), meaning nuclear purity of type one or type two nuclei will evolve with equal likelihood.

For diminishing returns to specialisation (0 < α < 1, 0 ≤ κ < 1), substituting 0 < α < 1 into $\frac{d^{2}W}{dx^{2}}<0$ (the condition for *x** to be a maximum) gives *px* ^α−2^ + (1 − *p*)(1 − *x*)^α−2^ > 0, which, given that 0 ≤ *p *≤* *1, is always satisfied, meaning *x** always represents a maximum. Because there is one maximum, *x** confers the global optimum fitness (*W*), and so represents an ESS. The maximum corresponds to genomic diversity (0<*x**<1) when the host plant environment is mixed (0 < *p *<* *1). Between‐individual selection therefore favours genomic diversity if there are diminishing returns to specialisation (0 < α < 1) and a mixed host plant environment (0 < *p *<* *1).

### APPENDIX 2: STABLE GENOMIC DIVERSITY

Equation 3 gives the change in the population mean nuclear proportion (*E*[*X*]) over one generation. The population mean nuclear proportion will not undergo further evolution if *E*[*X*]_*t*+1_ = *E*[*X*]_*t*_ = *E*[*X**]. By equating *E*[*X*]_*t*+1_ = *E*[*X*]_*t*_ and solving, we find this position (the *stationary distribution*) to be $E\left[X^{*}\right]=\mu +\frac{1-s}{s}\theta$ population with this average nuclear proportion (*E*[*X**]) will not evolve, but we now ask whether populations will evolve to this position from elsewhere (whether the stationary distribution is *absorbing*).

We perturb the equilibrium by a small positive value ε and see that rightward perturbations are restored if *E*[*X**] + ϵ > (*E*[*X**] + ϵ + θ)(1 − *s*) + *s*μ, and leftward perturbations are restored if *E*[*X**] − ϵ < (*E*[*X**] − ϵ + θ)(1 − *s*) + *s*μ. Substituting the equilibrium condition $E\left[X^{*}\right]=\mu +\frac{1-s}{s}\theta$ and simplifying generates ϵ > 0 in both cases, and so the population of individuals will evolve to this position (*E*[*X**]) regardless of its initial mean nucleus proportion (*E*[*X*]); it is an evolutionary end point.

We are interested in cases where populations maintain nuclear diversity within individuals. In principle, an intermediate mean population nuclear proportion (0 < *E*[*X**] < 1) could correspond to a mixture of genomically pure individuals, some with type one nuclei and others with type two. However, there is no diversifying selection in this model, and so nuclear diversity within the population corresponds to nuclear diversity within individuals (0 < *E*[*X**] < 1). 0 < *E*[*X**] always holds, because type two nucleus purity is never selected for. However, *E*[*X**] < 1 only holds for the condition given in Equation 4, which is the condition for stable genomic diversity.

### APPENDIX 3: COMPETING NUCLEI

In AM fungi, replicative differences between nuclei (θ) may be high, but in other organisms with multiple genomes, replicative synchrony (θ→0) might be well enforced. For example, other filamentous fungi (Basidiomycetes and Ascomycetes) can form dikaryons, in which replicative synchrony is often well enforced (by structures called *clamp connections* and *croziers*, respectively). Stable genomic diversity in these cases requires only that it provides some benefit to the individual (*s *>* *0).

Large genomic deletions may generate nuclei that are faster replicating as a result of their smaller genome, but non‐functional or deleterious to the individual. Between‐individual selection disfavours such nuclei (μ = 0), but we see that they can still coexist alongside functional nuclei if the between‐individual selection to purge the deleterious nuclei is (a) stronger that their replicative advantage within individuals ((1 − *s*)θ > *s*; this means that the equilibrium is a stable absorption point), and (b) not maximal, corresponding to lethal nuclei (*s *<* *1; this means that the absorption point is E[*X**] > 0). As predicted by this, deleterious ‘cheating’ nuclei have been observed in heterokaryotic fungi (Meunier *et al.* 2018; Bastiaans *et al.* 2016). A theoretical treatment of when such cheating nuclei will arise in the first place is a question for future study; here we are content to show that such nuclei, if they arise, can be maintained stably.

### APPENDIX 4: SIMULATION

We give further details regarding how nuclear replication, and individual dispersal, was modelled.

- Nucleus Replication Phase. Type 1 (*N*_*1*_) and type 2 (*N*_*2*_) nuclei replicate repeatedly, increasing exponentially: N_1_ (*t *+* *1) = (1 + *r*_*1*_) *N*_*1*_; N_2_ (*t *+* *1) = (1 + *r*_*2*_) *N*_*2*_, where the generational growth rate of type one nuclei (*r*_*1*_) exceeds that of type two nuclei (*r*_*1*_ > *r*_*2*_). An individual's generational change in nuclear proportion (*x*) is therefore given by: $x\left(t+1\right)=\frac{x(1+r_{1})}{x\left(r_{1}-r_{2}\right)+1+r_{2}}$
- Sporulation & Dispersal Phase. With probability *d*, an individual's offspring disperse and compete on a population scale with other dispersing offspring. There are *d*(*n*/*j*) spots available on each patch for dispersing offspring, and an individual with dispersing offspring reproduces into each of these spots with the probability given by their fitness (Equation 1) divided by the total fitness of all individuals with dispersing offspring. With probability (1 − *d*), an individual's offspring do not disperse and compete on the local patch with other non‐dispersing offspring for the (1 − *d*)(*n*/*j*) free spots. An individual with non‐dispersing offspring reproduces into each of these spots with the probability given by their fitness divided by the total fitness of all individuals with non‐dispersing offspring on the native patch.
