# Supplementary material for: Evolutionary maintenance of genomic diversity within arbuscular mycorrhizal fungi
Source: Ecol Evol. 2019 Feb 11;9(5):2425–35. doi: 10.1002/ece3.4834 (PMC6405528; doi:10.1002/ece3.4834)
Supplement: Supplementary file 1 [file ECE3-9-2425-s001.docx]

**Supplementary Information**

**Data S1: alternative lifecycle in the Competing Nuclei model**

We reformulate the Competing Nuclei model of the main text, assuming an alternative lifecycle where selection between individuals occurs at the spore stage, with nucleus replication following this as individuals mature. The order of within- and between-individual selection is therefore reversed relative to the model presented in the main text. The generational change in the population mean nuclear proportion is given by: E[X]*_t_*_+1_ = (sμ + (1-s) E[X]*_t_*) + θ. The absorption point becomes E[X*] = θ/s+μ, which corresponds to genomic diversity when s(1-μ)>θ. This qualitatively resembles the condition of the model presented in the main text (equation 4), capturing the opposing pulls of selection within and between individuals, except that the destabilising force of within-individual selection is here un-tempered by the strength of between-individual selection, as it occurs afterwards in the lifecycle (the right-hand side of the condition is θ rather than θ(1-*s*)).

**Data S2: more general capture of nucleus replication**

**in the Competing Nuclei model**

We extend the Competing Nuclei model of the main text to capture nucleus replication in a more general framework. Firstly, like in the original model, we assume a lifecycle in which within-individual selection precedes between-individual selection. In the original model, within-individual selection was captured by assuming that the nuclear proportion (which we denote here as X’) in an individual after a bout of within-individual selection can be given by X’=X+θ, where X is the nuclear proportion beforehand. This corresponds to a hypothetical lifecycle in which, every generation, a group of less competitive nuclei (N_2_) are replaced by a group more competitive nuclei (N_1_), with this group size constant with respect to the proportion of the two nuclei in individuals. This might occur if modular turnover is constrained, such that modules do not replicate freely.

However, the action of within-individual selection may depend on the current mean nuclear proportion (X). For example, if type one nuclei are nearly at fixation (X→1), further proportional increases in type one nuclei might be slight. We can model within-individual selection more generally: X’ = X + θ_1_ + θ_2_*X. Our formula for generational changes in nuclear proportion then becomes: *s*μ + (1-*s*) (E[*X*]_t_ + θ_1_ + E[*X*]_t_θ_2_) = E[X]_t+1_. Solving for E[*X*]_t_ = E[*X*]_t+1_ = E[*X**] and checking for stability, we find one absorption point at E[*X**] = (θ_1_ - *s*θ_1_ + *s*μ)/(*s* + (*s*-1)θ_2_), where μ and *s* respectively denote the optimum nuclear proportion and strength of between-individual selection. The mean corresponds to genomic diversity when (1-*s*)(θ_1_ + θ_2_)<*s*(1-μ). This condition qualitatively resembles the condition of the model presented in the main text (equation 4), capturing the opposing pulls of selection within and between individuals. The previous coefficient θ has been replaced the two coefficients, θ_1_ and θ_2_, which respectively capture X-independent and X-dependent generational shifts in nuclear proportion.

Higher orders of X are still required to capture other modes of nuclear replication (e.g. X’ = X + θ_1_ + θ_2_*X+ θ_3_*X^2^ +…), and the qualitative results of our abstract model are retained with arbitrary generalisations of nuclear replication. Similarly, we assumed that the response to between-individual selection (*s*) is constant and independent of nuclear proportion (X). This is contingent on the shape of the distribution of individuals and fitness values across different nuclear proportions. However, relaxing these assumptions, to allow the response to selection (*s*) to vary with the current nuclear proportion (X), does not change our qualitative findings. Furthermore, the results of the Competing Model are corroborated in the simulation model of the main text, in which within- and between-individual selection are modelled in a more biologically realistic scenario.

**Data S3: Additional Figures**

**P1**

**P2**

**O1**

**O2**

**O3**

**O4**

**G1**

**G2**

**Figure S1 | The theoretical problem of genomic diversity.** Two generations of AM fungi, G1 and G2, are represented. Hyphal networks (individuals) are represented by beige circles. Individuals bear nuclei, which can be different strains, as indicated by colour. Generation 1 (G1) consists of two parent individuals: P1, which has genomic diversity, and P2, which has genomic homogeneity. Genomic diversity leads to conflict and so P1 bears an evolutionary cost; P2 doesn’t bear this cost. P2 therefore has more asexual offspring (O2, O3 & O4) than P1 (O1), and so G2 is shifted towards genome purity as a result of between-individual selection. Due to competition within individuals (within-individual selection) or drift, O1 has a lower genomic diversity than its parent (P1), shifting G2 towards genome purity.


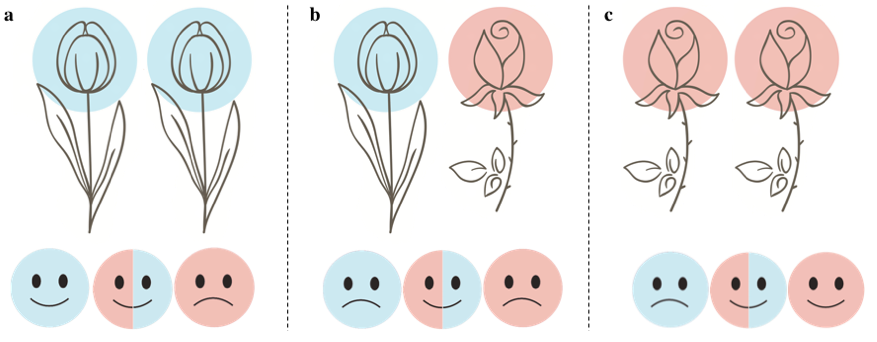


**Figure S2 | Hypothesis for the maintenance of genomic diversity.** The red and blue flowers represent two species of host plant. In environment (a) all host plants are the blue species; in (c) all are red; in (b) there is a mixture of blue and red. Host plants are connected to AM fungal networks (individuals), represented by faces, which contain multiple nuclei of two types. Individuals can contain solely nuclei that are specialised on red plants (red faces), solely nuclei that are specialised on blue plants (blue faces), or a mixture of nucleus types (red / blue faces). Mixed individuals grow well in all host plant environments (a-c) because they always contain some specialised nuclei. Pure individuals can only grow well when connected to their specialised host plant (blue face in (a); red face in (c)) but don’t grow well when there is a mixture of host plants (b). Genomic diversity may therefore be favoured in mixed host plant environments.


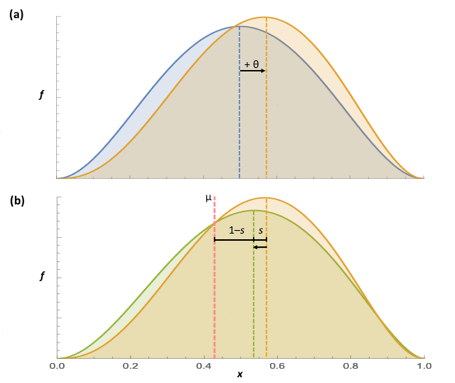


**Figure S3 | Competing Nuclei model set-up.** The distributions plot the frequency of AM fungal networks (individuals) with differing proportions of type one relative to type two nuclei (*x*) in a population. Each generation, the population undergoes within-individual selection (a), which increases the mean nuclear proportion (*E*[*X*]) by θ. The population then undergoes between-individual selection (b), which pulls the mean nuclear proportion (*E*[*X*]) towards the individual optimum nuclear proportion μ by some proportion given by the strength of between-individual selection (*s*). Individuals then reproduce asexually, which doesn’t change the mean nuclear proportion (*E*[*X*]), and the process is iterated. We ask if the two processes can lead to an equilibrium with genomic diversity (0<*E*[*X*]<1).


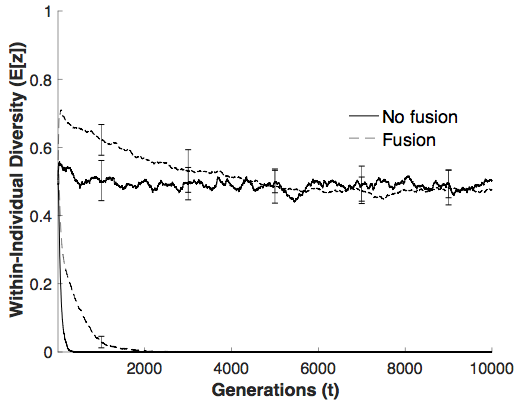


**(b) genomic diversity disfavoured**

**(a) genomic diversity favoured**

**Figure S4 | Effect of fusion on genomic diversity in the absence of within-individual selection.** Within-individual genomic diversity (*z**), in the absence of replicative differences between nuclei (*r*_1_=*r*_2_), is plotted as a function of time (*t*). Two scenarios are considered, when genomic diversity is: (a) favoured (α=0.3, *p*=0.3), and (b) disfavoured (α=1.1, *p*=0.6), by between-individual selection. The dashed lines assume fusion between individuals (*m*=0.2), and the solid lines assume no fusion (*m*=0). Under fusion, equilibrium is reached more slowly. This means that genomic diversity can be maintained in a non-equilibrium state over a longer time period, as is the case in (b). These results assumed *f*=0.005 (sporulation stochasticity), *d*=0.5 (dispersal). The plots represent the average results taken across 10 trials. Error bars, where plotted, show one standard deviation above and below the mean across these 10 trials.


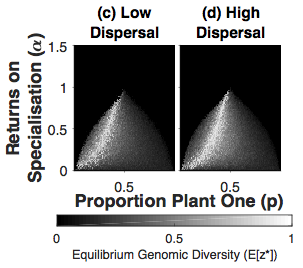

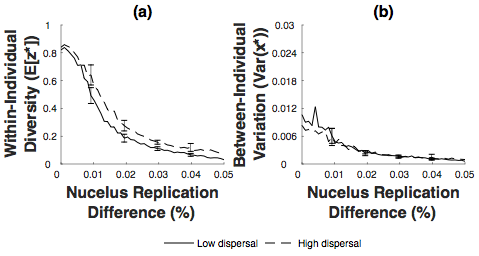


**Figure S5 | Effect of dispersal on genomic diversity.** The within-individual genomic diversity (a), and between-individual variation in nuclear proportion (b), is plotted against the nuclear replicative advantage of type one nuclei (*r_1_*-*r_2_*/*r_2_*) (α=0.8, p=0.5, *d*=0.5, *r_2_*=0.3, *r_1_* is varied). The different lines represent different degrees of dispersal (low: *d*=0.5; high: *d*=1). Dispersal does not significantly affect between-individual variation, but can nevertheless slightly increase within-individual diversity, because it increases the effective population size of the population, and hence the efficacy of between-individual selection. The plots represent the average results taken across 10 trials. Error bars, where plotted, show one standard deviation above and below the mean across these 10 trials. (c) and (d) plot the full range of between-individual selection, from decelerating to accelerating returns on plant specialisation (α, y axis), and from a plant two to a plant one dominated environment (*p*, x axis). Equilibrium genomic diversity is slightly greater when dispersal is high, across a large range of between-individual selection. These results assumed *f*=0.005 (sporulation stochasticity) and *m*=0 (fusion).
