# Supplementary material for: Evolutionary maintenance of genomic diversity within arbuscular mycorrhizal fungi
Source: Ecol Evol. 2019 Feb 11;9(5):2425–35. doi: 10.1002/ece3.4834 (PMC6405528; doi:10.1002/ece3.4834)
Supplement: Supplementary file 2 [file ECE3-9-2425-s002.docx]

**Appendix**

**Appendix 1: Between-Individual Selection**

Equation 2 gives the nuclear proportion corresponding to a stationary point (*x**). It can be seen by inspection of Equation 2 that there is always, and only, one sensical *x** value (one that lies in the range 0≤*x*≤1) for any combination of parameter values. The stationary point (*x**) could represent a maximum (if $\frac{d^{2}W}{dx^{2}}<0$), minimum ($\frac{d^{2}W}{dx^{2}}>0$) or inflection point (if $\frac{d^{2}W}{dx^{2}}=0$). If it is a maximum, then *x** represents the individual-favoured nuclear proportion (the ESS). If it is a minimum or inflection point, then the individual-favoured nuclear proportion (the ESS) will be found at a boundary of *x*=0 or *x*=1.

We examine the form of the stationary point for the different ranges of the shape parameter α and baseline fitness κ. When there are increasing returns to specialisation (α>1) and nuclear proportions affect fitness (0≤κ<1), substituting α>1 into $\frac{d^{2}W}{dx^{2}}>0$ (the condition for *x** to be a minimum) gives *px^α–2^* + (1 – *p*)(1 – *x*)^α–2^ > 0, which, given that 0≤*p*≤1, is always true. *x** therefore always represents a minimum when returns are increasing. When returns to specialisation are linear (α=1, 0≤κ<1) we find that $\frac{d^{2}W}{dx^{2}}=0$, and so *x** always represents an inflection point.

We ask what ESS will arise when *x** represents a minimum or inflection point. Given that there is only one equilibrium solution *x** for each set of parameter values, it must be the case that *W*(*x*) is maximal at either *x*=0 or *x*=1. It is maximal at *x*=1 if *W*(*x*=0)<*W*(*x*=1) is satisfied. Evaluating this shows that this is true for *p*>0.5. Conversely, *W*(*x*) is maximised at *x*=0 when *p*<0.5. When *W*(*x*=0)=*W*(*x*=1), which is the case when *p*=0.5, individuals can maximize fitness with either of two strategies, and individuals may assume either *x*=0 or *x*=1 at equilibrium. So, when returns to specialisation are increasing or linear (α≥1), the ESS is positioned at nuclear purity, and the nucleus type that is chosen is the one that grows better with the most common plant host. In the special case where nuclear proportions have no effect on fitness (κ=1), substitution of κ=1 gives $\frac{d^{2}W}{dx^{2}}=0$ and *W*(*x*=0)=*W*(*x*=1), meaning nuclear purity of type one or type two nuclei will evolve with equal likelihood.

For diminishing returns to specialisation (0<α<1, 0≤κ<1), substituting 0<α<1 into $\frac{d^{2}W}{dx^{2}}<0$ (the condition for *x** to be a maximum) gives *px*^α–2^ + (1 – *p*)(1 – *x*)^α–2^ > 0, which, given that 0≤*p*≤1, is always satisfied, meaning *x** always represents a maximum. Because there is one maximum, *x** confers the global optimum fitness (*W*), and so represents an ESS. The maximum corresponds to genomic diversity (0<*x**<1) when the host plant environment is mixed (0<*p*<1). Between-individual selection therefore favours genomic diversity if there are diminishing returns to specialisation (0<α<1) and a mixed host plant environment (0<*p*<1).

**Appendix 2: Stable Genomic Diversity**

Equation 3 gives the change in the population mean nuclear proportion (*E*[*X*]) over one generation. The population mean nuclear proportion will not undergo further evolution if *E*[*X*]*_t_* _+ 1_ = *E*[*X*]*_t_* = *E*[*X**]. By equating *E*[*X*]*_t_* _+ 1_ = *E*[*X*]*_t_* and solving, we find this position (the *stationary distribution*) to be $E\left[ X^{*} \right]=\mu+\frac{1-s}{s}\theta$. A population with this average nuclear proportion (*E*[*X**]) will not evolve, but we now ask whether populations will evolve to this position from elsewhere (whether the stationary distribution is *absorbing*).

We perturb the equilibrium by a small positive value and see that rightward perturbations are restored if *E*[*X**] + ϵ > (*E*[*X**] + ϵ + θ)(1 – *s*) + *s*μ, and leftward perturbations are restored if *E*[*X**] – ϵ < (*E*[*X**] – ϵ + θ)(1 – *s*) + *s*μ. Substituting the equilibrium condition $E\left[ X^{*} \right]=\mu+\frac{1-s}{s}\theta$ and simplifying generates ϵ > 0 in both cases, and so the population of individuals will evolve to this position (*E*[*X**]) regardless of its initial mean nucleus proportion (*E*[*X*]); it is an evolutionary end point.

We are interested in cases where populations maintain nuclear diversity within individuals. In principle, an intermediate mean population nuclear proportion (0 < *E*[*X**] < 1) could correspond to a mixture of genomically pure individuals, some with type one nuclei and others with type two. However, there is no diversifying selection in this model, and so nuclear diversity within the population corresponds to nuclear diversity within individuals (0 < *E*[*X**] < 1). 0 < *E*[*X**] always holds, because type two nucleus purity is never selected for. However, *E*[*X**] < 1 only holds for the condition given in Equation 4, which is the condition for stable genomic diversity.

**Appendix 3: Competing Nuclei**

In AM fungi, replicative differences between nuclei (θ) may be high, but in other organisms with multiple genomes, replicative synchrony (θ→0) might be well enforced. For example, other filamentous fungi (Basidiomycetes and Ascomycetes) can form dikaryons, in which replicative synchrony is often well enforced (by structures called *clamp connections* and *croziers*, respectively). Stable genomic diversity in these cases requires only that it provides some benefit to the individual (*s*>0).

Large genomic deletions may generate nuclei that are faster replicating as a result of their smaller genome, but non-functional or deleterious to the individual. Between-individual selection disfavours such nuclei (μ=0), but we see that they can still coexist alongside functional nuclei if the between-individual selection to purge the deleterious nuclei is (a) stronger that their replicative advantage within individuals ((1-*s*)θ>*s*; this means that the equilibrium is a stable absorption point), and (b) not maximal, corresponding to lethal nuclei (*s*<1; this means that the absorption point is E[*X**]>0). As predicted by this, deleterious ‘cheating’ nuclei have been observed in heterokaryotic fungi (Meunier *et al.* 2018; Bastiaans *et al.* 2016). A theoretical treatment of when such cheating nuclei will arise in the first place is a question for future study; here we are content to show that such nuclei, if they arise, can be maintained stably.

**Appendix 4: Simulation**

We give further details regarding how nuclear replication, and individual dispersal, was modelled.

1. **Nucleus Replication Phase.** Type one (*N_1_*) and type two (*N_2_*) nuclei replicate repeatedly, increasing exponentially: N_1_ (*t* + 1) = (1 + *r_1_*) *N_1_*; N_2_ (*t* + 1) = (1 + *r_2_*) *N_2_*, where the generational growth rate of type one nuclei (*r_1_*) exceeds that of type two nuclei (*r_1_>r_2_*). An individual’s generational change in nuclear proportion (*x*) is therefore given by: $x\left( t+1 \right)=\frac{x(1+r_{1})}{x{(r}_{1}-r_{2})+1+r_{2}}$
2. **Sporulation & Dispersal Phase.** With probability *d*, an individual’s offspring disperse and compete on a population scale with other dispersing offspring. There are *d*(*n*/*j*) spots available on each patch for dispersing offspring, and an individual with dispersing offspring reproduces into each of these spots with the probability given by their fitness (Equation 1) divided by the total fitness of all individuals with dispersing offspring. With probability (1 – *d*), an individual’s offspring do not disperse and compete on the local patch with other non-dispersing offspring for the (1 – *d*)(*n*/*j*) free spots. An individual with non-dispersing offspring reproduces into each of these spots with the probability given by their fitness divided by the total fitness of all individuals with non-dispersing offspring on the native patch.
